# Live Attenuated *B. pertussis* BPZE1 Rescues the Immune Functions of Respiratory Syncytial Virus Infected Human Dendritic Cells by Promoting Th1/Th17 Responses

**DOI:** 10.1371/journal.pone.0100166

**Published:** 2014-06-26

**Authors:** Ilaria Schiavoni, Giorgio Fedele, Adriano Quattrini, Manuela Bianco, Corinna Schnoeller, Peter J. Openshaw, Camille Locht, Clara M. Ausiello

**Affiliations:** 1 Dipartimento di Malattie Infettive, Parassitarie ed Immunomediate, Istituto Superiore di Sanità, Rome, Italy; 2 Centre for Respiratory Infection, National Heart and Lung Institute, Imperial College London, London, United Kingdom; 3 INSERM U1019, Lille, France; 4 Centre National de la Recherche Scientifique UMR8204, Lille, France; 5 Centre d’Infection et Immunité de Lille, Institut Pasteur de Lille, Lille, France; 6 Univ Lille Nord de France, Lille, France; University of Iowa, United States of America

## Abstract

Respiratory Syncytial virus (RSV) is the leading cause of acute lower respiratory tract viral infection in young children and a major cause of winter hospitalization. *Bordetella pertussis* is a common cause of bacterial lung disease, affecting a similar age group. Although vaccines are available for *B. pertussis* infection, disease rates have recently increased in many countries. We have therefore developed a novel live attenuated *B. pertussis* strain (BPZE1), which has recently undergone a successful clinical phase I trial. In mice, BPZE1 provides protection against disease caused by respiratory viral challenge. Here, we analyze the effect of BPZE1 on antiviral T cell responses induced by human monocyte-derived dendritic cells (MDDC). We found that BPZE1 influences antiviral immune responses at several levels, enhancing MDDC maturation, IL-12p70 production, and shifting T cell cytokine profile towards a Th1/Th17 pattern. These data were supported by the intracellular signaling analysis. RSV infection of MDDC caused MyD88-independent STAT1 phosphorylation, whereas BPZE1 activated MyD88-dependent signaling pathways; co-infection caused both pathways to be activated. These findings suggest that BPZE1 given during infancy might improve the course and outcome of viral lung disease in addition to providing specific protection against *B. pertussis* infection.

## Introduction

Respiratory Syncytial virus (RSV), a single-stranded RNA virus in the *Paramyxoviridae* family, is the leading cause of acute lower respiratory tract infections in infants and the single major cause of childhood hospitalization in the developed world [1]. Approximately 60% of all children are infected with RSV in the first year of life, increasing to 90% by their second year of life [2]. Despite the presence of serum antibody, RSV re-infects throughout life. Globally, RSV is thought to cause almost 34 million cases per year of acute lower respiratory tract infection in children under 5 years of age, 10% of them being severe. Infantile bronchiolitis is associated with recurrent wheezing in older children [3]–[5]. It is thought that RSV bronchiolitis represents an overactive host immune response to infection [6], and there is still no safe and effective RSV vaccine for human use. Indeed, the human trials of formaldehyde-inactivated RSV vaccine in 1966–1967 caused disastrous worsening of disease and death in infants during subsequent natural RSV infection [7], [8].

A live attenuated *B. pertussis* vaccine strain, named BPZE1, has been developed as a vaccine candidate against whooping cough [9]. It has successfully completed a phase I safety trials (http://www.child-innovac.org) [10]. BPZE1 induces strong Th1 responses in mice, and long-lasting protective immunity against *B. pertussis* after a single nasal administration. It has proven safe, even in neonatal and immunodeficient animals [11], [12]. Moreover, Li et al. [13], recently, reported that nasal administration of BPZE1 provides effective protection against lethal challenge with highly virulent mouse-adapted H3N2 and H1N1 (A/PR/8/34) influenza A viruses by controlling influenza virus-mediated inflammation [13].

Dendritic cells (DC) constantly monitor the lungs for pathogens or foreign antigens and play a key role in the initiation of inflammatory responses at the mucosal surface. Local DC become activated during RSV infection [14], [15]. They acquire viral antigens, undergo maturation and migrate to the lung-draining lymph nodes, where they present peptide epitopes to prime naive T cells [15].

We have previously demonstrated that BPZE1 is able to mature and activate human monocyte-derived DC (MDDC) and to induce the release of pro-inflammatory and regulatory cytokines. In addition, BPZE1-primed MDDC drive a mixed Th1/Th17 polarization and induce functional T suppressor cells [16].

RSV and *B. pertussis* can naturally co-infect infants, but children with co-infection appear to experience a milder disease [17]. Schnoeller and colleagues demonstrated that BPZE1 protects against RSV disease in a respiratory viral challenge mouse model via an IL-17-dependent mechanism [18]. We therefore tested the capacity of live attenuated BPZE1 to rescue RSV-induced immune responses in the pre-clinical human MDDC model and explored the intracellular signaling pathways involved. We found BPZE1 co-infected with RSV enhances maturation of MDDC and drives the T cells polarization to a putative protective Th1/Th17 response.

## Materials and Methods

### Ethics Statement

This study was conducted according to the principles of the Declaration of Helsinki [19]. Peripheral blood was collected from healthy blood donors at the Centro Trasfusionale Policlinico Umberto I, University La Sapienza blood bank (Rome, Italy, courtesy of Dr. Girelli). The blood samples were handed over anonymously. None of the authors were involved in collecting the blood samples or had access to patient identifying information.

All blood donors provided written informed consent for the collection of samples and subsequent analysis. Blood samples were processed anonymously, the materials once used for the experiments were then destroyed, genetic research or interventions that include genome were not included in the research protocol. This study was conducted within the project ChildINNOVAC, in compliance with European Commission FP7 ethical rules, a specific IRB for this research was not approached.

### Preparation of RSV stocks

Plaque-purified human RSV (type A2 strain from the ATCC) was grown in HEp-2 cells [20] with Dulbecco’s modified Eagle’s medium (DMEM, Life Technologies Invitrogen, Paisley, UK), containing heat inactivated 10% Foetal Bovine Serum (FBS; Hyclone Laboratories, South Logan, UT). Mock-infected HEp-2 cells were similarly processed to generate the mock control stock preparation. Viral titers were determined by plaque assay, as described [20].

### Bacterial strain and growth conditions

The BPZE1 strain is derived from *B. pertussis* Tohama I. It is devoid of pertussis toxin enzymatic activity, lacks dermonecrotic toxin and is deficient in tracheal cytotoxin release as previously described [9]. BPZE1 was grown on charcoal agar plates supplemented with 10% defibrinated sheep blood (Biotec, Grosseto, Italy) for 48 h at 37°C. Bacteria were then collected and suspended in 2 ml phosphate-buffered saline (PBS), and the concentration was estimated by measuring the optical density (OD) at 600 nm. The suspensions were adjusted to a final concentration of 10^9^ colony-forming units (CFU)/ml. The bacterial concentration was then checked retrospectively by CFU evaluation of the final bacterial suspension.

### Purification and culture of MDDC

Human monocytes were purified from peripheral blood lymphocytes were isolated by using Ficoll gradients (lympholyte-H; Cedarlane, Burlington, Ontario). CD14 cells were purified by positive sorting through anti-CD14 mAb-conjugated magnetic microbeads (Miltenyi Biotec, Bergisch Gladbach, Germany) and cultured in 75 cm^2^ flasks (Costar, Corning Life Sciences, Tewksbury, MA) in RPMI 1640 medium (Life Technologies Invitrogen), supplemented with heat-inactivated 10% lipopolysaccharide-free FBS, 1 mM sodium pyruvate, 0.1 mM nonessential amino acids, 2 mM L-glutamine, 25 mM HEPES, 100 U/ml penicillin, 100 µg/ml streptomycin (all from EuroClone, Milan, Italy) and 0.05 mM 2-βmercaptoethanol (Sigma Chemicals; St. Louis, MO) in the presence of human recombinant GM-CSF (25 ng/ml; R&D Systems, Minneapolis, MN) and IL-4 (25 ng/ml; R&D Systems) [21]. After 6 d, immature MDDC were washed and analyzed by cytofluorimetry for the expression of the surface markers CD1a, CD14, CD83 and CD38. MDDC were used in the experiments if >80% CD1a and <10% CD14 positive cells.

### Infection of MDDC with RSV and/or BPZE1

MDDC (10^6^ cell/ml) were incubated with RSV at multiplicity of infection (MOI) of 1 and/or with BPZE1, at bacteria- to- cells ratio of 100∶1. After 2 h at 37°C cells were extensively washed in the presence of polymyxin B at 5 µg/ml (Sigma Chemicals) to kill extracellular bacteria and incubated in 1 ml of complete medium at 37°C, 5% CO_2_ [16]. In all experiments mock control was added to MDDC. At 24 h post infection, MDDC were harvested for immunophenotypic analysis, and the supernatants were collected for cytokine measurement by ELISA.

### RSV RT-PCR

RNA was extracted from MDDC cultures following RSV or mock infection, and the concentration and quality of the RNA were determined by spectrophotometry. Reverse transcription was carried out as previously described [20] using 1 µg of total RNA for cDNA synthesis. qPCR specific for the RSV L gene was performed using 900 nM forward primer (5′-GAACTCAGTGTAGGTAGAATGTTTGCA-3′), 300 nM reverse primer (5′-TTCAGCTATCATTTTCTCTGCCAAT-3′), and 175 nM probe (5′-6-carboxyfluorescein-TTTGAACCTGTCTGAACATTCCCGGTT-6-carboxytetramethylrhodamine-3′). Copy numbers were determined from standard curves of pCDNA3 containing a fragment of the RSV L-gene [20].

### Apoptosis detection

Apoptosis of MDDC was detected by using the Annexin V-FITC Apoptosis Detection Kit (eBioscience, San Diego, CA) [22]. Briefly, MDDC were treated with stimuli for either 24 or 48 h, as indicated, harvested and double-stained with FITC-conjugated annexin V and propidium iodide, according to the manufacturer’s protocol. Cells were analyzed by flowcytometry in a FACScan (BD Biosciences, San Jose, CA) using CellQuest software. Data are expressed as % of positive cells.

### Immunophenotypic analysis

Cells were washed, suspended in PBS containing 3% FBS and 0.09% NaN_3_, and incubated with a panel of fluorochrome-conjugated mAbs (obtained from BD Biosciences) specific for MDDC differentiation/maturation (anti- CD80, CD83 and CD38) [16]. Isotype-matched Abs were used as negative controls. Cells were analyzed with a FACScan (BD Biosciences). Fluorescence data were reported as percentages of positive cells when the treatment induced the expression of the marker in cells that were negative (CD83). Median fluorescence intensity (MedFI) was used when the treatment increased the expression of the marker in cells that were already positive (CD80 and CD38).

### Determination of cytokine levels by ELISA

To measure cytokine production by infected MDDC, supernatants were collected, and IL-10, IL-12p70 and IL-23 production were assessed by ELISA [16]. The lower detection limits were: IL-10∶3.9 pg/ml, IL-12p70∶5.0 pg/ml, IL-23∶20.0 pg/ml. Cytokines in the supernatants from polarized T cells were assayed by ELISA specific for IFNγ, IL-5 and IL-17. The lower detection limits were: IFNγ: 8.0 pg/ml, IL-5∶3.0 pg/ml, IL-17∶15.0 pg/ml. OD obtained was measured with a 3550-ultraviolet Microplate Reader (BioRad, Philadelphia, PA) at 450 nm. All ELISA kits were from Quantikine, R&D Systems, except for IL-23 (Bender Med System, Burlingame, CA).

### Isolation of T lymphocytes and MDDC-T cell allogeneic MLR

CD3 T cells were purified from PBMC by negative sorting with magnetic beads (Pan T-cell Kit, Miltenyi Biotec). Purity of cell preparations was higher than 95%, as assessed by cytofluorimetric staining. RSV-, BPZE1- or double- infected MDDC were cultured in MLR with fresh allogeneic T cells (3 x 10^5^) at different MDDC/T cell ratios in 48-well cell culture plates for 6 d. Cell proliferation was measured by Bromodeoxyuridine (BrdU) (BD Biosciences) incorporation [16]. Briefly, BrdU was added to the MDDC and T cells at 3 µg/ml final concentration on d 3 of culture. Cells, collected on d 6, were fixed in 0.5% paraformaldehyde, permeabilized and stained for intracellular BrdU by direct immunofluorescence with a FITC-conjugated anti-BrdU mAb with DNase (BD Biosciences). Cells were examined by flow cytometry, and T cell proliferation was evaluated as percentage of BrdU-positive cells.

### Polarization of T lymphocytes

To evaluate T-lymphocyte polarization, experiments were performed using fresh allogeneic CD3 T cells purified by negative sorting with the PanT isolation kit (Miltenyi Biotec). Purity of cell preparations was higher than 95%, as assessed by cytofluorimetric staining. MDDC were treated with RSV and/or BPZE1 for 48 h, washed extensively, and then co-cultured (0.5×10^5^) with CD3 T cells (0.5×10^6^) in 24-well plates (Costar, Corning Life Sciences). On d 6, rIL-2 at 50 U/ml (PeproTech, Rocky Hill, NJ) was added to the cultures. On d 12, supernatants were harvested for cytokine measurement by ELISA [16].

### Determination of protein kinase phosphorylation by Western blot analysis

MDDC were mock infected or challenged with BPZE1, RSV, BPZE1/RSV for 20 min or 2 h and then lysed using RIPA buffer as previously described [23]. For the 24 h time point, the cells were infected for 2 h, then extensively washed in the presence of polymyxin B (5 µg/ml), incubated in complete medium at 37°C, 5% CO_2_ for 24 h and then lysed using RIPA buffer. Immunoreactive proteins were detected by incubating blots with anti-phosphorylated proteins overnight at 4°C. Rabbit polyclonal IgG anti-p-p44/42 mitogen-activated protein kinases (MAPK) (Thr202/Tyr204, ERK1/2), anti-p-p38 MAPK (Thr180/Tyr182), anti-p-I kappa B alpha (IkBα) (Ser32), anti-p-stat1 (Tyr701) were from Cell Signaling Technology, Inc. (Danvers, MA). Rabbit anti-β actin was from Hyclone Laboratories (South Logan, UT). Blots were washed in Tris-buffered saline 0.1% Tween-20, incubated with horse-radish-peroxidase (HRP)-conjugated goat anti-rabbit IgG (Bio-Rad Laboratories, Hercules, CA) to reveal phosphorylated proteins or HRP-conjugated goat anti-mouse IgG (GE Healthcare, Little Chalfont, UK) to reveal control proteins. The proteins were developed with the enhanced chemiluminescence ECL reagents from Pierce (Rockford, IL). Densitometry was performed on scanned immunoblot imaging using Model GS-700 Imaging Densitomer (Biorad, Laboratories) and Multi-Analyst software to obtain OD. The relative OD for each band was calculated by normalizing the OD for the corresponding β actin OD.

### mRNA Cytokine Expression by TaqMan Real-Time RT-PCR Analysis

To measure cytokine mRNA expression, TaqMan Real-time RT-PCR analysis was used (Applied biosystems, Foster City, CA). Total RNA was extracted from infected MDDC at different time points, and reverse transcription was carried out as previously described [21], [24]. TaqMan assays were performed according to manufacturer’s instructions with an ABI 7700 thermocycler (Applied biosystems). Human β actin was analyzed as an internal control, and mRNA transcript levels were expressed as fold increase normalized to β actin. Fold changes in gene expression levels were calculated by comparison with the gene expression in mock sample, which were assigned an arbitrary value of 1.

### Statistical analysis

Statistical analysis was performed using the GraphPad Prism software (San Diego, California, US). For the overall comparison of median values, a statistical analysis, using Fridman test, was performed. For post-test pairwise comparisons, Wilcoxon matched pairs test, adjusting for 6 multiple comparisons (0.05/6 = 0.0083), was then used. *P*≤0.0083 was considered statistically significant.

## Results

### Impact of BPZE1 on phenotypic maturation and cytokine production of RSV-infected MDDC

To determine the conditions of RSV infection, MDDC were infected for 2 h with RSV at two MOI: 0.5 and 1 plaque forming units/cell. The infection was monitored by measuring the RSV L gene transcripts as index of viral copies. MDDC were more efficiently infected at a MOI of 1 compared to a MOI of 0.5, both at 24 h and 48 h (0.27E+04 vs 0.08E+04 viral copies/µg RNA, and 8.00E+04 vs 5.40E+04 viral copies/µg RNA, respectively). Therefore, the MOI of 1 was chosen for the subsequent experiments.

In a previous study we found that 2 h of MDDC infection by BPZE1 at a bacteria/cell ratio of 100∶1 followed by 24 h of culture allowed for optimal MDDC maturation and that, at this ratio, BPZE1 promoted significant resistance of MDDC to apoptosis, spontaneously occurring when the cells were not stimulated [16]. To assess whether RSV and BPZE1 co-infections affected cell viability, necrosis and apoptosis were measured in MDDC infected with BPZE1, RSV, or both. Mock treated- MDDC were included as control. We found that whereas RSV, in agreement with previous studies [25], enhanced the levels of apoptosis with respect to mock treated-MDDC, protection from spontaneous apoptosis was observed in double infected MDDC (23.6±8.7 vs 33.0±8.3% of Annexin V positive cells, mean values of 4 experiments performed at 48 h in BPZE1/RSV- vs RSV- treated MDDC, respectively. Apoptosis rate of mock treated MDDC in the same experiments was 17.4±4.4% of positive cells).

The transition from an immature to a mature stage endows DC with the capacity to couple innate to adaptive immunity. To evaluate this transition in our experimental conditions, MDDC were infected with BPZE1, RSV or both. Mock treated- MDDC were included as control. The phenotypic maturation of MDDC was evaluated by monitoring the surface expression of CD80, CD83 and CD38. CD80 co-stimulatory molecule is involved in APC functions of MDDC, whereas CD83 is one of the best-known surface markers for fully mature MDDC [26], and CD38 is an early maturation marker induced by microbial molecules and cytokines such as IFNγ produced during the innate immune response [22], [27].

**Figure 1:**
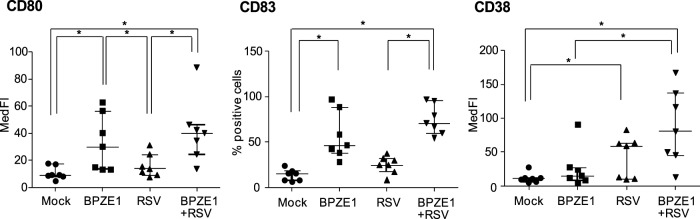
Impact of BPZE1 on phenotypic maturation of RSV-infected MDDC. MDDC were treated with BPZE1 (bacteria/cell ratio 100∶1), RSV (MOI of 1) or both. After 24 h, cells were analyzed for the indicated surface markers associated with a mature phenotype. Mock-infected cells were used as control. Fluorescence data are reported as median fluorescence intensity (MedFI) for CD80 and CD38, and as percentage of positive cells for CD83. Values are expressed as medians with interquartile range of seven independent experiments performed with MDDC obtained from different donors. Statistical significant differences are indicated by * (*P*≤0.0083).

RSV infection induced a modest up-regulation of the markers analyzed, with a significant CD38 increase, as compared to mock treated cells. When MDDC were treated simultaneously with BPZE1 and RSV, the induction of a fully mature phenotype was observed, with higher CD38 expression as compared to BPZE1-challenged MDDC (Figure 1).

**Figure 2:**
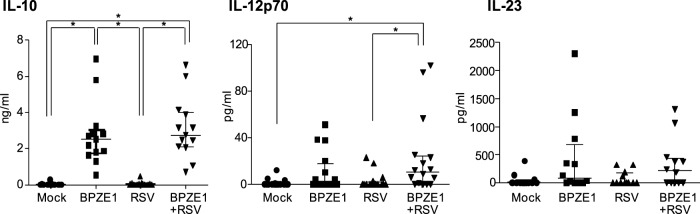
Impact of BPZE1 on IL-10, IL-12p70 and IL-23 production of RSV-infected MDDC. MDDC were treated with BPZE1 (bacteria/cell ratio of 100∶1), RSV (MOI of 1) or double infected with BPZE1 and RSV. Mock-infected cells were used as control. After 24 h, cytokines released in the culture media were measured by ELISA. Values are expressed as medians with interquartile range from 13 (IL-10 and IL-23) and from 15 (IL-12p70) independent experiments performed with MDDC obtained from different donors and expressed as ng/ml (IL-10) or pg/ml (IL-12p70 and IL-23). Statistical significant differences are indicated by * (*P*≤0.0083).

DC maturation leads to the production of several key inflammatory and immunoregulatory cytokines, which rule the development of cell-mediated and humoral immune responses [28]. In order to determine the impact of BPZE1, RSV, or BPZE1/RSV double infection on the release of cytokines involved in the polarization of Th responses, we measured IL-10, a cytokine with a predominant immunosuppressive function, IL-12p70, a pro-inflammatory cytokine important in Th1 polarization, and IL-23, involved in Th17 polarization [28], in the supernatants of infected MDDC. Figure 2 shows that BPZE1 induced the production of high levels of IL-10, low levels of IL-12p70, (measurable only in a minority of the samples analyzed) and appreciable levels of IL-23, as compared to mock-treated MDDC. RSV-infected MDDC did not induce the production of measurable amounts of IL-10 and induced barely detectable levels of IL-12p70 and IL-23. However, when MDDC were co-infected with RSV and BPZE1 IL-10 and IL-23 were produced at levels similar to those induced by BPZE1 alone. Interestingly, IL-12p70 secretion was increased in double infected cultures with respect to BPZE1-, RSV- infected cells and mock- treated MDDC, reaching a statistical significance as compared to RSV- and mock- treated MDDC.

### Impact of BPZE1 on RSV-induced allogeneic T cell proliferation and Th response

**Figure 3:**
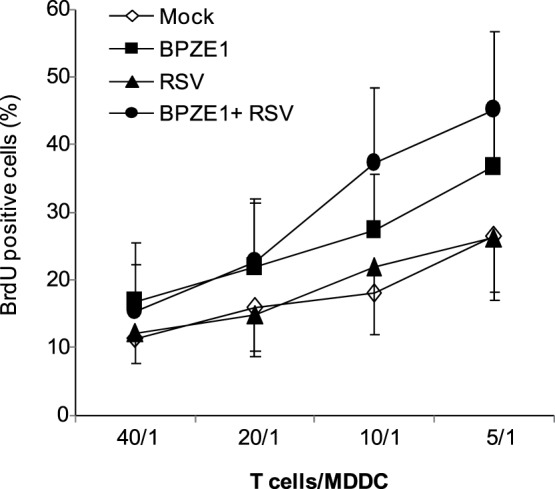
Impact of BPZE1 on RSV-induced allogeneic T cell proliferation. MDDC were infected with RSV (MOI of 1), BPZE1 (bacteria/cell ratio of 100∶1), or both. Mock-infected cells were added as control. After 48 h, MDDC were co-cultured with allogeneic purified T cells at increasing T cells/MDDC ratios (5/1, 10/1, 20/1, 40/1) for 6 d. Results are reported as percentages of BrdU positive cells (mean ± SEM of 4 independent experiments performed with MDDC obtained from different donors).

A key function of mature DC is antigen presentation to T lymphocytes and polarization of the immune response [29]. The antigen-presenting ability was evaluated by measuring the proliferation of T lymphocytes co-cultured for 6 days with allogeneic MDDC. As shown in Figure 3, MDDC stimulated with BPZE1 efficiently induced T cell proliferation. In contrast, RSV-infected MDDC induced only basal levels of T cell proliferation, in agreement with previous studies [30], [31]. When BPZE1 and RSV were added simultaneously, MDDC very efficiently induced allogeneic T cell proliferation, with an increasing trend as compared to the proliferation induced by BPZE1-treated MDDC.

**Figure 4:**
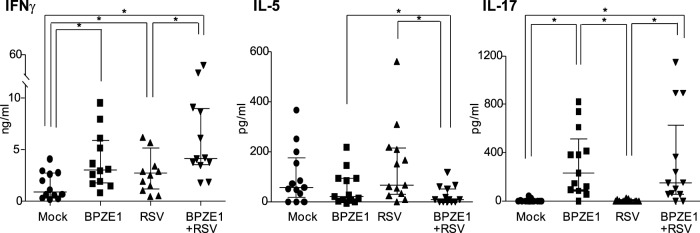
Impact of BPZE1 on RSV-induced T lymphocyte polarization. MDDC were treated with BPZE1 (bacteria/cell ratio of 100∶1), RSV (MOI of 1) or both. Mock-infected cells were added as control. After 48 h, treated MDDC were cultured with purified allogeneic CD3 T cells. On day12, supernatants were collected, and IFN-γ (Th1), IL-5 (Th2) and IL-17 (Th17) were measured by ELISA. Values are expressed as medians with interquartile range of 13 independent experiments performed with MDDC obtained from different donors and expressed as ng/ml (IFNγ) or pg/ml (IL-5 and IL-17). Statistical significant differences are indicated by * (*P*≤0.0083).

We next investigated the capacity of BPZE1-, RSV-, or double-infected MDDC to polarize the T cell responses. Polarization experiments were performed by co-culturing purified allogeneic CD3 T lymphocytes with BPZE1-, RSV- or BPZE1/RSV-infected MDDC and measuring the levels of three key cytokines of polarized Th responses: IFNγ, landmark of Th1 immune responses, IL-5 produced by Th2 effectors and IL-17, mediator of Th17 responses [28]. BPZE1-treated MDDC induced a mixed Th1/Th17 polarization, with high levels of IFNγ and IL-17 but basal levels of IL-5, as expected from previous observations [16]. RSV-infected MDDC, instead, showed a Th1 polarization, as evidenced by consistent levels of IFNγ and clearly did not induce IL-17 production. The addition of BPZE1 to RSV-infected MDDC caused a marked and significant increase of IL-17 and IFNγ, compared to RSV-infected and mock-treated MDDC. At the same time IL-5 induction was statistically decreased with respect to RSV and BPZE1- infected MDDC (Figure 4). Overall, when BPZE1 was added to RSV, the MDDC-mediated polarization shifted towards a clear Th1/Th17 profile.

### RSV- and BPZE1-induced signaling pathways and gene profile in MDDC

To better understand the activation of pro-inflammatory cytokines in our system, we characterized the pathways triggered by RSV in infected MDDC and the influence exerted by BPZE1. We analyzed the phosphorylation of the transcription factor STAT1, pivotal in the regulation of the MyD88-independent pathway [32], the phosphorylation of IκBα, a process required for the activation of NF-κB, and the phosphorylation of p44/p42 (ERK1/2) and p38, MAPKs family members involved in the MyD88-dependent pathway [33].

**Figure 5:**
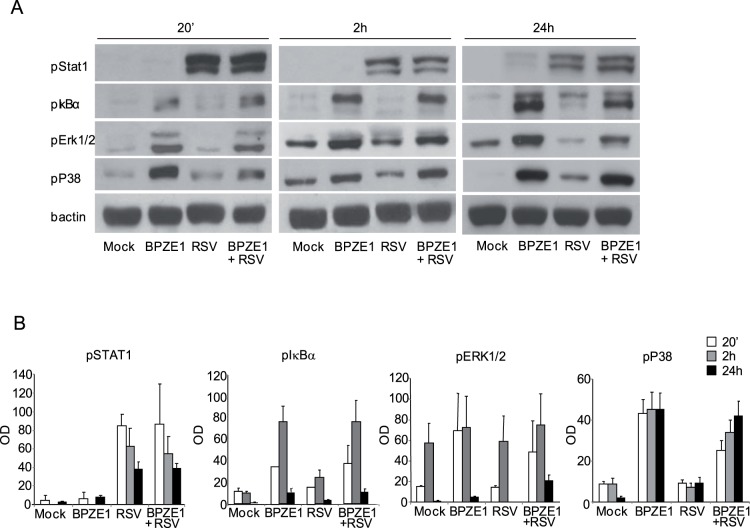
RSV- and BPZE1-induced signaling pathways in MDDC. **A**: Total cell lysates were prepared from MDDC treated with BPZE1 (100∶1), RSV (MOI of 1) or both at the indicated time-points. Mock-infected cells were added as control. Proteins were resolved by 10% SDS-PAGE and Western blot analysis was performed to detect the phosphorylation of STAT1, IkBα, ERK1/2 and p38. Human phosphorylated β actin was used to normalize the results. Data are from one representative of three independent experiments performed with MDDC obtained from different donors. **B**: Histograms represent means of the relative optical density of phosphorylated proteins from three independent experiments. Error bars represent SEM.

Figure 5 shows that BPZE1 treatment induced phosphorylation of ERK1/2, p38, and IkBα, similar to the results obtained with the virulent *B. pertussis* in a previous study [34], but not of STAT1. Conversely, RSV induced in MDDC phosphorylation of STAT1 at all time points analyzed, but not of the other proteins tested (except trace amount of phosphorylated p38 at 24 h, in Figure 5 panel A). These results indicate that, in our system, RSV triggers mainly a MyD88-independent pathway while BPZE1 a MyD88-dependent pathway. When MDDC were simultaneously infected with BPZE1 and RSV the phosphorylation of all proteins tested was observed. Overall these data indicate that BPZE1 and RSV trigger two different signaling pathways leading to a strong activation of MDDC.

The MyD88-dependent pathway is known to be responsible for the activation of several pro-inflammatory genes, including those encoding for IL-6 and the p40 subunit of IL-12p70 [35], [36]. On the other hand, the MyD88-independent signaling is responsible for the activation of intracellular pathways that, through the activation of IRF3, drive the production of IFNβ and, consequently, the expression of IFN-inducible genes such as those coding for CCL5 (also known as RANTES) and the p35 subunit of IL-12p70 [36], [37], both of which have been shown to play an important part in the response to RSV infection in mice [18].

**Figure 6:**
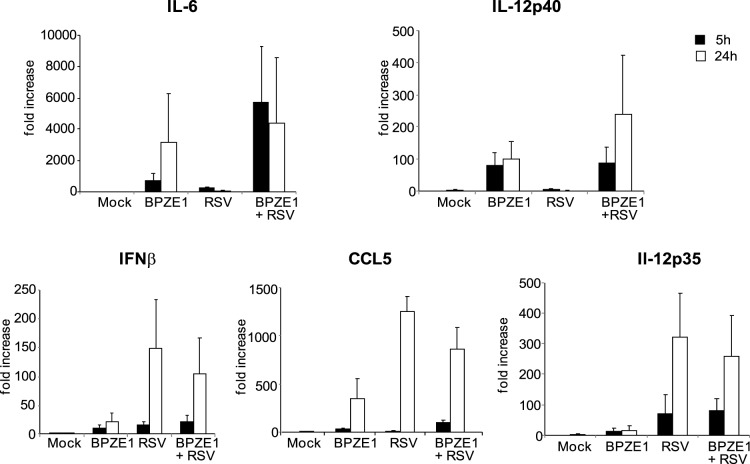
RSV- and BPZE1-induced IL-6, IFNβ, CCL5, IL-12p40 and IL-12p35 gene expression in MDDC. MDDC were treated with BPZE1 (100∶1), RSV (MOI of 1) or both. Mock-infected cells were added as control. Total RNA was extracted at the indicated time points. Kinetics of mRNA expression for p40 and p35 subunits of IL-12p70, IL-6, IFNβ and CCL5 was evaluated by real-time quantitative RT-PCR. The mRNA transcripts were normalized with respect to the endogenous reference (human β actin) sample. Data were expressed as fold increase (mean ± SEM of four experiments) with respect to mock-treated cells at 5 h.

To confirm the differential triggering of MyD88-dependent or -independent pathways by BPZE1 and RSV in MDDC, we analyzed the expression levels of the above mentioned genes in our experimental conditions (Figure 6). As expected, BPZE1 was able to induce the expression of the genes coding for IL-6 and the IL-12p40 subunit, which were not induced by RSV. In contrast, RSV triggered the expression of the genes coding for IFNβ, CCL5 and the IL-12p35 subunit, which are ruled by MyD88-independent signaling and were not (or marginally for CCL5) induced by BPZE1. When double infections were performed, we observed the expression of all the genes tested, albeit with a decrease for IFNβ compared to RSV treatment alone. Overall these data confirm those obtained by Western Blot analysis and indicate the simultaneous activation of two different pathways in double infected MDDC.

## Discussion

It is known that RSV can infect human DC *in vitro* and activate MDDC [25], [38]–[40]. The present study shows that the live attenuated *B. pertussis* vaccine candidate BPZE1 can infect human MDDC and modulate the immune response to RSV by (i) enhancing their maturation; (ii) enhancing, IL-12p70 production and (iii) switching their polarization ability to a Th1/Th17 pattern.

Despite to the basal levels of CD80 and CD83 induction, RSV significantly enhanced surface expression of CD38 on MDDC. CD38 is an ectoenzyme and a marker of maturation and activation involved in many MDDC functions, including migration, survival and the induction of Th1 polarized immune responses [22], [27]. Enhanced CD38 expression is also functionally involved in IL-12p70 induction [27]. In our study, RSV alone did not induce release of IL-12p70, but double-infected MDDC released more IL-12p70 than MDDC primed with BPZE1 alone. Further studies are needed to evaluate if the up-regulation of CD38 may be involved in the enhanced release of IL-12p70.

Other cytokines are crucially involved in the orchestration of the adaptive immune response such as IL-10 and IL-23. We observed an increase of these cytokines in double-infected cells suggesting a positive effects of infection with RSV and BPZE1. Mature DC possess remarkable antigen-presenting abilities and activate the proliferation of T cells in the lymph nodes. RSV infection of DC affects the ability to activate the proliferation of T cells [40], [41], possibly because of RSV’s ability to suppress the formation of immunological synapses between RSV-infected DC and T cells (as demonstrated in murine DC, ref [25]), or the secretion of regulatory cytokines released by RSV-infected DC, such as IFN-λ and -α, involved in the suppression of T cells [42]. Our study confirms the inability of RSV-infected MDDC to activate allogeneic T cell proliferation and, at the same time, evidences the property of BPZE1 to overcome the inhibition of T cell responses mediated by RSV.

Our data show a tendency by RSV-primed MDDC to activate a Th1 response, although the Th2 component was not increased as compared to that induced by mock-primed MDDC. In this regard, considering the relative high level of IL-5 induced by mock treated MDDC, our data suggest that this *ex vivo* system is already Th2 oriented and that the capacity to induce this polarization could be inherently driven by immature or low-mature MDDC [43], [44]. BPZE1/RSV co-infected MDDC shifted the Th response towards a Th1/Th17 profile, with IFNγ levels higher than those induced by RSV-MDDC (*P = *0.0049). This shift in Th polarization is of particular interest considering that the clearance of RSV infection in healthy individuals needs the activation of IFNγ-secreting CD8 and CD4 T cells, and that the unbalance of Th1 and Th2 response is associated with severe forms of RSV-induced diseases [45], [46].

Animal studies have associated Th2 responses with increased RSV-induced pulmonary pathology (38, 39). Depletion of the Th2-associated cytokine IL-4 prior to acute RSV infection has been reported to decrease lung inflammation and mucus production [47]. By contrast, Th1 responses may be essential for promoting protective immunity, and depletion of the Th1 polarizing cytokine IL-12p70 results in increased production of the Th2-associated cytokine IL-13, along with increased airway resistance, pulmonary inflammation and mucus production [48]. It is therefore possible that, in infected individuals, BPZE1 could protect against RSV-mediated detrimental Th2-associated responses and ensure the generation of putative protective Th1/Th17 responses. These data are in agreement with recent evidences in mice where BPZE1 did not exacerbate the inflammatory response to infection with RSV, but rather provided considerable protection. This non-specific effect was long-lasting and depended on the cytokine IL-17 [18].

The role of IL-17 in RSV disease appears to be highly dependent on context [49]; in mice, IL-17A is reported to inhibit airway reactivity induced by RSV infection during allergic airway inflammation [50]. In a human study, an increase of local IL-17 occurs during the recovery of children hospitalized with RSV [51].

RSV is able to activate different receptors of the innate immune response, including TLRs [52], [53] and *B. pertussis* has been shown to trigger TLR2 and TLR4 in human cells and mice [24], [34], [54]–[56].

We hypothesized that the effects exerted by BPZE1 on RSV-infected MDDC could be mediated by the activation of a different intracellular signaling pathway that may act co-operatively and foster the production of IL-12p70, antigen-presentation activity, and the capacity to drive Th1 cell expansion. Our data show that BPZE1 is able to activate MyD88 signaling in MDDC but does not activate STAT1 phosphorylation associated with the MyD88-independent pathway. In contrast, RSV activates STAT1 phosphorylation but not the MyD88-dependent pathway. As a result, co-infection activates both pathways. This was confirmed by transcriptional analyses of gene expression induced by both the MyD88-dependent and -independent pathways. Interestingly, the expression of both subunit genes of IL-12p70 was induced by double infections, and a significant increment of IL-12p70 levels released by MDDC was detected (Figure 2, *P* = 0.0068 BPZE1+RSV vs RVS; *P* = 0.0024 BPZE1+RSV vs Mock).

Overall our results indicate that the live attenuated *B. pertussis* vaccine candidate BPZE1 can rescue the RSV-induced immune response in human MDDC, enhances the maturation state of the MDDC, increases IL-12p70 production and induces a Th1/Th17 cytokine profile. These data were supported by the results of the intracellular signaling pathway.

The present study gives further support for the use of BPZE1 in infants and young children not only as a vaccine against pertussis (http://www.child-innovac.org) [10], but also for possible benefits during viral infections, in the acceleration of postnatal immune maturation and in protection against allergic disorders, as it may play an important role as an adjuvant that can drive a protective Th1/Th17 response, important in RSV infection and in other pathological disorders.
